# microRNA-184 Induces a Commitment Switch to Epidermal Differentiation

**DOI:** 10.1016/j.stemcr.2017.10.030

**Published:** 2017-11-30

**Authors:** Sara Nagosa, Friederike Leesch, Daria Putin, Swarnabh Bhattacharya, Anna Altshuler, Laura Serror, Aya Amitai-Lange, Waseem Nasser, Edith Aberdam, Matthieu Rouleau, Sudhir G. Tattikota, Matthew N. Poy, Daniel Aberdam, Ruby Shalom-Feuerstein

**Affiliations:** 1 Department of Genetics and Developmental Biology, The Rappaport Faculty of Medicine, Technion - Israel Institute of Technology, Haifa 31096, Israel; 2 University Paris Diderot, Sorbonne Paris Cité, Paris 75475, France; 3 INSERM U976, Hôpital St-Louis, Paris 75010, France; 4 CNRS, LP2M, UMR7370, Faculté de Médecine, Nice, France; 5 Université Nice Sophia Antipolis, Nice, France; 6 Max Delbrueck Center for Molecular Medicine, Robert Roessle Strasse 10, Berlin 13125, Germany

**Keywords:** microRNA, miR-184, miRNA-184, K15, FIH1, notch, stem cells, epidermis, hair follicle, cornea

## Abstract

miR-184 is a highly evolutionary conserved microRNA (miRNA) from fly to human. The importance of miR-184 was underscored by the discovery that point mutations in miR-184 gene led to corneal/lens blinding disease. However, miR-184-related function *in vivo* remained unclear. Here, we report that the miR-184 knockout mouse model displayed increased p63 expression in line with epidermal hyperplasia, while forced expression of miR-184 by stem/progenitor cells enhanced the Notch pathway and induced epidermal hypoplasia. In line, miR-184 reduced clonogenicity and accelerated differentiation of human epidermal cells. We showed that by directly repressing cytokeratin 15 (K15) and FIH1, miR-184 induces Notch activation and epidermal differentiation. The disease-causing miR-184^C57U^ mutant failed to repress K15 and FIH1 and to induce Notch activation, suggesting a loss-of-function mechanism. Altogether, we propose that, by targeting K15 and FIH1, miR-184 regulates the transition from proliferation to early differentiation, while mis-expression or mutation in miR-184 results in impaired homeostasis.

## Highlights

• The levels of miR-184 are enhanced upon exit from the stem cell niche
• miR-184 represses epidermal proliferation and stemness
• miR-184 represses K15 and FIH1, both of which prevent epidermal differentiation
• mutated miR-184 failed to repress K15 and FIH1 and failed to induce differentiation

## Graphical Abstract

**Figure:**
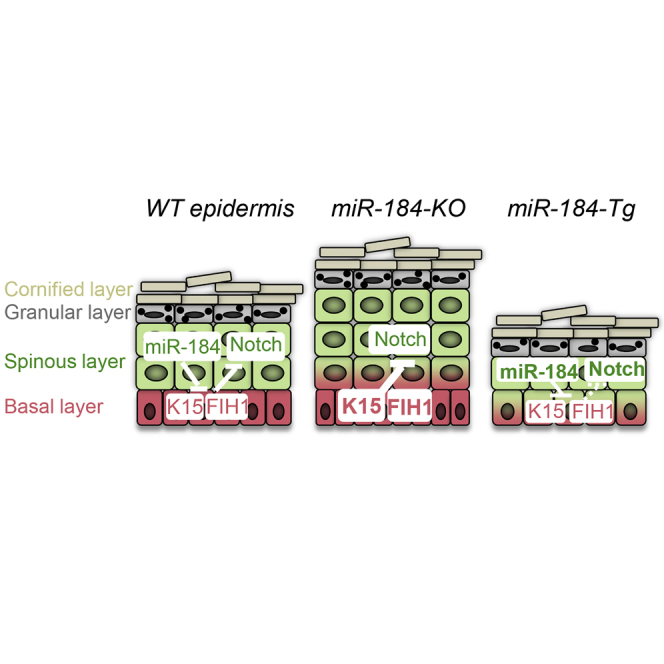

## Teaser

Using new genetic mouse models and study of human epidermal cells, Nagosa et al. show that miR-184 regulates epidermal proliferation and commitment to differentiation. The authors discovered that miR-184 directly represses K15 and FIH1, which are important for the maintenance of stemness phenotype.

## Introduction

The skin and cornea serve as a barrier that is protecting our body against exterior insults. The outermost layer of both tissues is a stratified squamous epithelium that is continuously regenerated by stem cells (SCs) during homeostasis (Blanpain and Fuchs, 2006, O'Callaghan and Daniels, 2011). It was traditionally believed that the interfollicular epidermis (IFE) is renewed by SCs or a pool of progenitors that reside in the basal layer, a niche that provides localized signals to maintain stemness (Fuchs, 2009, Morrison and Spradling, 2008, Ordonez and Di Girolamo, 2012). Accordingly, cells that exit the niche and transit to the spinous epidermal layer undergo commitment to the terminal differentiation. Similar to the IFE, hair-follicle SCs (HFSCs) and limbal SCs (LSCs) that regenerate the corneal epithelium are located in discrete niches and engage similar differentiation programs (Amitai-Lange et al., 2015, Cotsarelis et al., 1989, Cotsarelis et al., 1990, Di Girolamo et al., 2015).

The classical dogma describes epidermal SCs as slow-cycling cells that are rare in the niche, surrounded by ten fast-dividing but short-lived progenitor cells (Mascre et al., 2012, Potten et al., 1974). This deterministic paradigm can explain the heterogeneity of basal epidermal cells *in vivo* and *ex vivo*; however, against this dogma, there are no specific markers that label specifically slow-cycling SCs. An alternative stochastic model suggests that the entire basal layer is occupied by equipotent fast-dividing progenitor cells that compete for niche factors for survival (Clayton et al., 2007, Rompolas et al., 2016, Bacman et al., 2013). In light of this controversy, it would be important to clarify the mechanisms of SC regulation and the sharp switch from proliferative to post-mitotic compartment, which is only partially understood.

p63 plays a key role in the maintenance of epidermal proliferation and stemness (Blanpain and Fuchs, 2007, Senoo et al., 2007) while Notch represses p63 and cell proliferation and activates a cell epidermal differentiation program (Rangarajan et al., 2001, Nguyen et al., 2006). The antagonizing role of p63 and Notch is common to the differentiation programs of epidermis, hair follicle, and cornea. In line, p63-null mice fail to develop all types of stratified epithelia (Mills et al., 1999, Shalom-Feuerstein et al., 2011, Yang et al., 1999) while Notch1-deficient mice display epidermal hyperplasia and aberrant differentiation (Rangarajan et al., 2001). However, while the function and molecular cues controlled by p63 and Notch are well described in the literature, very little is known regarding the signals that regulate p63 and Notch expression or activity.

MicroRNAs (miRNAs) play a role in regulating diverse biological processes, including SC maintenance and differentiation. However, accumulating reports suggest that most miRNA knockout mouse strains displayed mild or no phenotype, unless stress was applied (Park et al., 2010). In line, only few miRNA encoding genes were associated with human genetic diseases. miR-184 is a remarkably evolutionary conserved miRNA, which was shown to be essential for the commitment of embryonic stem cells into corneal-epithelial cells (Shalom-Feuerstein et al., 2012). miR-184 was shown to be involved in various biological processes, including germline development in the fly (Iovino et al., 2009), neural fate (Liu et al., 2010, Nomura et al., 2008), cell proliferation, and migration (Yu et al., 2008, Yu et al., 2010). Interestingly, four different point mutations in miR-184 were linked with lens/corneal dystrophy and blindness (Farzadfard et al., 2016, Hughes et al., 2011, Lechner et al., 2013, Iliff et al., 2012). However, the different functions of miR-184 *in vivo* under homeostasis and the etiology of miR-184-related eye pathology remained to be investigated.

Here, we report that the level of miR-184 is significantly elevated in committed cells of the epidermis, hair follicle, and corneal epithelium. By generating loss-of-function and gain-of-function mouse models, we found that miR-184 controls the balance between epidermal cell proliferation and differentiation. The molecular mechanism involves direct repression of K15 and FIH1, induction of Notch pathway, and cell differentiation.

## Results

### Compartmentalized Expression Pattern of miR-184

**Figure 1:**
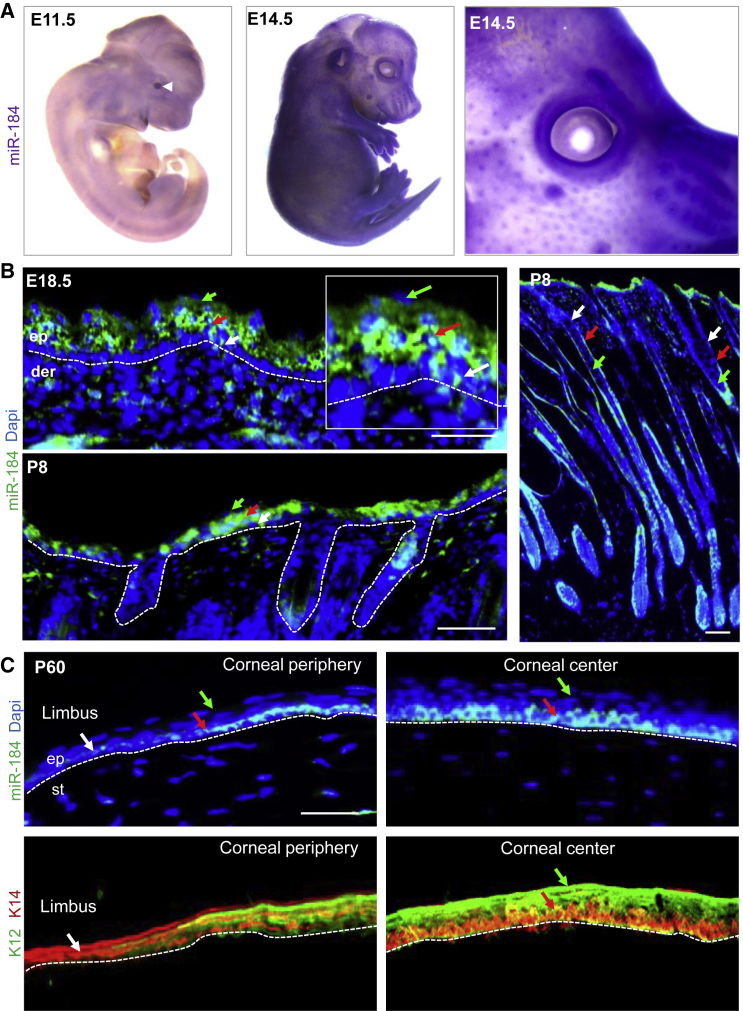
Expression Profile of miR-184 in the Murine Skin and Cornea *In situ* hybridization was performed on whole embryos (A) or tissue sections (B and C) of wild-type mice on the indicated embryonic day (see also Figures 5B and 5C). (A) Signal of miR-184 was evident in the developing lens at E11.5 (arrowhead) while at E14.5, the levels of miR-184 increased in the epidermis and hair follicles. At E18.5 and P8 (B), most epidermal basal cells expressed low levels of miR-184 (white arrow), while miR-184 was highly expressed in the spinous layer (red arrow) but not in terminally differentiated cells (green arrow). Inset in (B) is the enlarged epidermal region shown for E18.5. In the hair follicle (B, right image), miR-184 was not detected in the bulge SC niche (white arrow), expressed by early committed inner root sheet (red arrow), and matrix cells but not in terminally differentiated hair cells (green arrow). (C) Mouse cornea at P60 showed a similar pattern of low signal of miR-184 in the SC niche (limbus, white arrow, defined K14 staining of the adjacent section in the lower panel), early committed corneal basal epithelial cells expressed high levels (red arrow), while terminally differentiated corneal supra-basal cells (green arrow, K12-positive, compare with lower panel) were negative. The dashed lines indicate the dermal-epidermal (B) and corneal stromal-epithelial (C) junction. Scale bars, 50 μm. der, dermis; ep, epithelium; st, stroma.

At murine embryonic day 11.5 (E11.5), miR-184 was highly expressed in the developing lens (Figure 1A, arrowhead) while from E14.5–18.5 to postnatal stages, a significant signal was detected in the developing epidermis and hair follicles (Figures 1A and 1B). Low or no signal was found in the epidermal basal layer cells at E18.5 and postnatal day 8 (P8) (Figure 1B, high magnification, white arrow). However, a clear signal was found in the spinous layer (red arrow) and no signal was evident in late terminally differentiated cells (green arrow) (Figures 1B and S1). Likewise, miR-184 was not expressed in the hair-follicle SC niche (bulge) but was detected in early committed outer root sheath cells (ORS) and matrix cells and not expressed by terminally differentiated hair shaft cells (Figure 1B, see also Figures 5B and 5C). In contrast to the epidermis, corneal stratification begins after birth, and SC niche function was demonstrated by lineage tracing of 2-month-old mice (Amitai-Lange et al., 2015, Di Girolamo et al., 2015). At P60, miR-184 was expressed at low levels in the SC niche (limbus, white arrow), highly induced in early committed basal layer peripheral and central corneal epithelium (red arrow) but not by terminally differentiated (K12-expressing) corneal supra-basal cells (green arrow) (Figure 1C). To further explore the specificity of miR-184 expression in epidermal cells we performed *in situ* hybridization and real-time PCR analysis. We confirmed that miR-184 is expressed in the epidermis of wild-type and not miR-184-deficient epidermis (Figures S2E–S2F), miR-184 is expressed by primary human and mouse keratinocytes (KCs) and repressed by anti-miR antagonist (Figure S2G) and is expressed in heart, epidermal, and corneal cells but not in fibroblasts (Figure S2H). Altogether, miR-184 displays a common expression pattern in the differentiation program of the epidermis, hair follicle, and corneal epithelium; it is low or absent in the SC compartment, high in early committed cells, and absent in terminal differentiated cells.

### Modulation of *miR-184* Expression in Mice Results in Abnormal Epidermal Proliferation

To investigate the importance of miR-184 *in vivo*, a miR-184 knockout (*KO*) mouse model carrying a conditional loss-of-function allele was generated. These mice were crossed with a ubiquitously expressed Cre transgene (*ROSA26-Cre*) to generate miR-184-heterozygous (*HT*) mice (Figures S2A–S2B). Genotyping, quantitative real-time PCR, and *in situ* hybridization analyses confirmed that miR-184 is not expressed by *KO* mice (Figures S2C, S2E, and S2F). Although the number of *HT* and *KO* newborn pups was lower than expected from the Mendelian ratio, this difference was not statistically significant (chi-squared test p value 0.2518, n = 255, Figure S2D). No gross phenotype was observed in *HT* and *KO* mice, which macroscopically appeared comparable with wild-type (WT) control mice and were fertile. Eye phenotypes will be described elsewhere.

**Figure 2:**
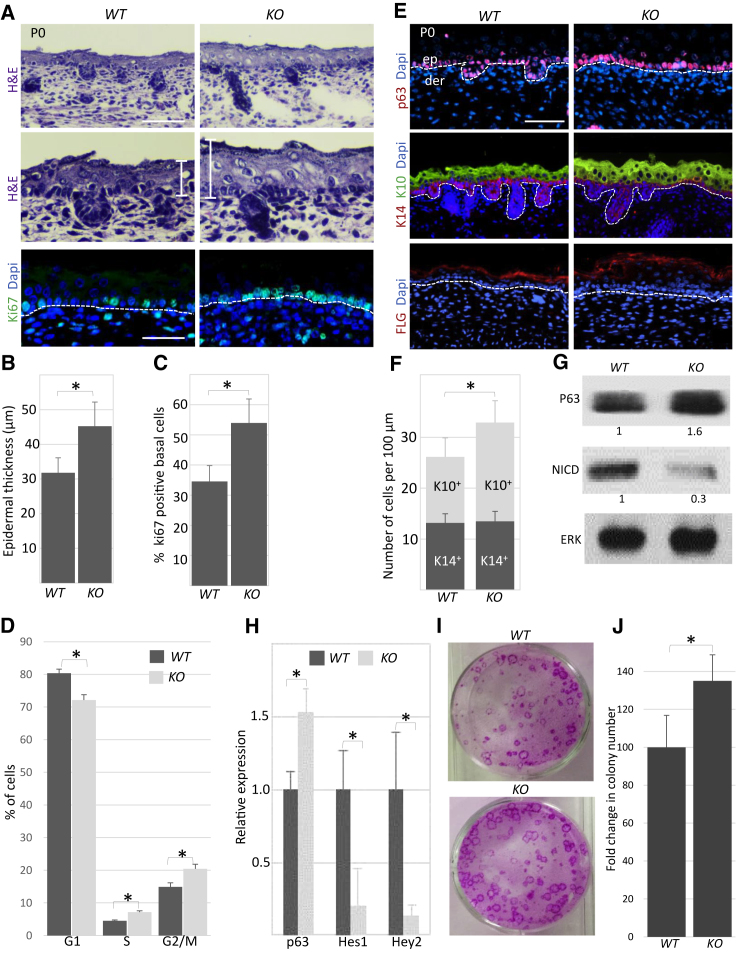
miR-184-Deficient Mice Displayed Increased Proliferation and Epidermal Hyperplasia (A–C and E) Paraffin sections of the head of newborn mice of the indicated genotypes were used for histology staining with H&E (A) or for immunofluorescent staining of the indicated proteins (A and E). Lower histology pictures in (A) are enlargements of the upper panels. Quantification of epidermal thickness (B) and the percentage of Ki67-positive basal layer cells (C) were performed as detailed in the Experimental Procedures. (D) Cell-cycle analysis of cells that were freshly isolated from the epidermis of the indicated genotypes. (F) Quantification of K14- and K10-positive cells was performed as detailed in the Experimental Procedures. (G) Protein lysates were prepared from the epidermis of newborn mice of the indicated genotypes and used for western blot analyses of the indicated proteins. Values represent densitometry analysis of three independent experiments, as detailed in the Experimental Procedures. (H) RNA extraction of the epidermis of newborn mice of the indicated genotypes was used for quantitative real-time PCR analyses of the indicated genes. (I and J) KCs were isolated from the epidermis of newborn mice of the indicated genotypes, subjected to clonogenicity test, and visualized by Rhodamine staining (I). Quantification by computerized analysis is shown in (J). Data shown are means ± SD from three independent experiments. ^∗^p < 0.05 statistically significant by Student's t test. The dashed lines (A and E) indicate the dermal-epidermal junction. Quantification of western blot analysis from three independent experiments (p < 0.05) is shown at the bottom of each panel. Scale bars are 50 μm. de, dermis; ep, epidermis.

**Figure 3:**
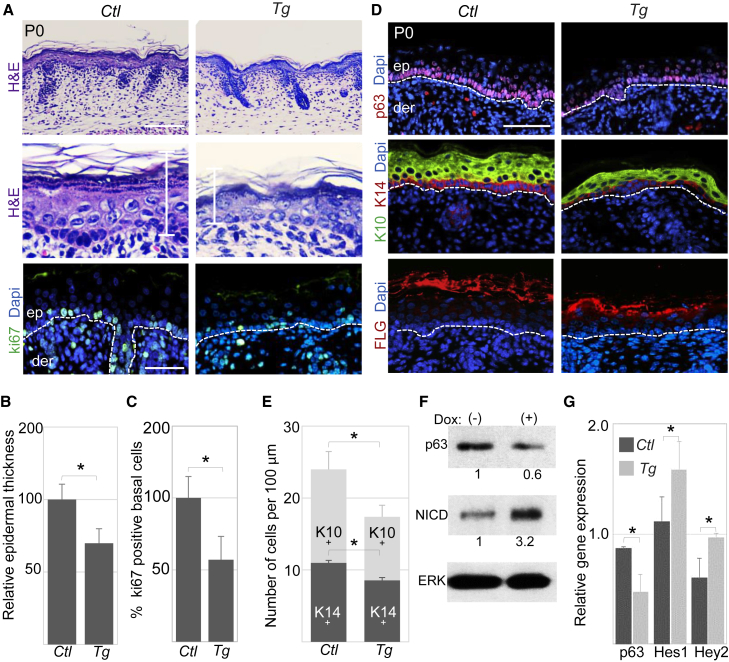
Forced Overexpression of miR-184 Attenuated Proliferation and Induced Epidermal Hypoplasia (A–D) Paraffin sections of the head of newborn mice of the indicated genotypes were used for histology staining with H&E (A) or for immunofluorescent staining of the indicated proteins (A and D). Lower histology pictures in (A) are enlargements of the upper panels. Quantification of epidermal thickness (B) and the percentage of Ki67-positive basal layer cells (C) were performed as detailed in the Experimental Procedures. (E) Quantification of K14- and K10-positive cells was performed as detailed in the Experimental Procedures. (F) Primary KCs were extracted from newborn miR-184 transgenic mice, induced with Dox (+) or vehicle (−) for 48 hr, and subjected to western blot analysis for p63, NICD, or ERK as loading control. Values represent densitometry analysis of three independent experiments, as detailed in the Experimental Procedures. (G) RNA extraction of the epidermis of newborn mice of the indicated genotypes was used for quantitative real-time PCR analyses of the indicated genes. Data shown are means ± SD from three independent experiments. ^∗^p < 0.05 statistically significant by Student's t test. The dashed lines (A and D) indicate the dermal-epidermal junction. Quantification of western blot analysis from three independent experiments (p < 0.05) is shown at the bottom of each panel. Scale bars are 50 μm. de, dermis; ep, epidermis.

Histology of newborn mice showed a significant increase in epidermal thickness in *KO* mice compared with WT counterparts (Figures 2A and 2B). In line, an increased number of proliferating Ki67-positive cells was observed in miR-184-null epidermis, indicating that miR-184 represses cell proliferation (Figures 2A and 2C). In agreement, ablation of miR-184 resulted in a reduction in the percentage of epidermal cells in G1 and an increase in cells found to be in S and G2/M phase (Figures 2D, S3A, and S3B). Although miR-184 is expressed by hair-follicle cells, no change was found in hair-follicle morphology and cell proliferation. To investigate whether the thickening of the epidermis is associated with a specific layer, we performed staining of K14 (basal layer), K10 (spinous layer), and Filaggrin (cornified layer). All epidermal layers were present in the miR-184-null mice; however, the spinous K10-positive cell layer was significantly thicker (∼30% increase) (Figures 2E and 2F). The changes in proliferation and thickening of the spinous layer raised the possibility that the switch from the basal layer to the spinous layer, which is known to be controlled by p63 (Senoo et al., 2007) and Notch (Nguyen et al., 2006, Rangarajan et al., 2001), is interrupted in miR-184 knockout skin. Indeed, the levels of p63 increased while the levels of active Notch (NICD) and its downstream target genes, Hes1 and Hey2, decreased (Figures 2E, 2G, and 2H). Since miR-184 is low or not expressed by basal cells (Figure 1B), the effect on the basal layer may involve a non-cell-autonomous mechanism. However, low signal was evident sometimes and a cell-autonomous effect could not be excluded (see Discussion). The expression of miR-184 in early committed spinous cells, together with its impact on proliferation and the Notch pathway, suggests that miR-184 may serve as a molecular switch to epidermal differentiation. In line, primary KCs that were subjected to a clonogenicity test have shown increased colony-forming potential, compared with their wild-type counterparts (Figures 2I and 2J). To test this hypothesis further, a transgenic mouse strain allowing tetracycline-inducible expression of miR-184 under the promoter of K14 has been produced (Figures S4A and S4B). Administration of the tetracycline analog doxycycline (Dox) from E12 efficiently induced miR-184 only in double-transgenic animals (*K14-rtTA;miR-184*^*Tg*^, referred to hereafter as Tg) (Figure S4C). Since no phenotypic difference was found between other genotyped littermates (WT, *K14-rtTA*, or *miR-184*^*Tg*^), *K14-rtTA* was used as a control in the next experiments. Newborn Tg pups that were induced by Dox displayed significant epidermal hypoplasia coupled to reduced cell proliferation compared with control pups (Figures 3A–3C). This was mainly due to thinning of the K10-positive spinous layer (Figures 3D and 3E). Importantly, the levels of p63 decreased while the Notch pathway was enhanced in Tg animals (Figures 3D, 3F, and 3G). Altogether, these data indicate that miR-184 tilts the balance between Notch and p63 in the epidermis, represses epidermal cell proliferation, and may be involved in early steps of epidermal commitment to differentiation.

### miR-184 Modulates Colony Formation Capacity and Accelerates Differentiation of Human Keratinocytes

**Figure 4:**
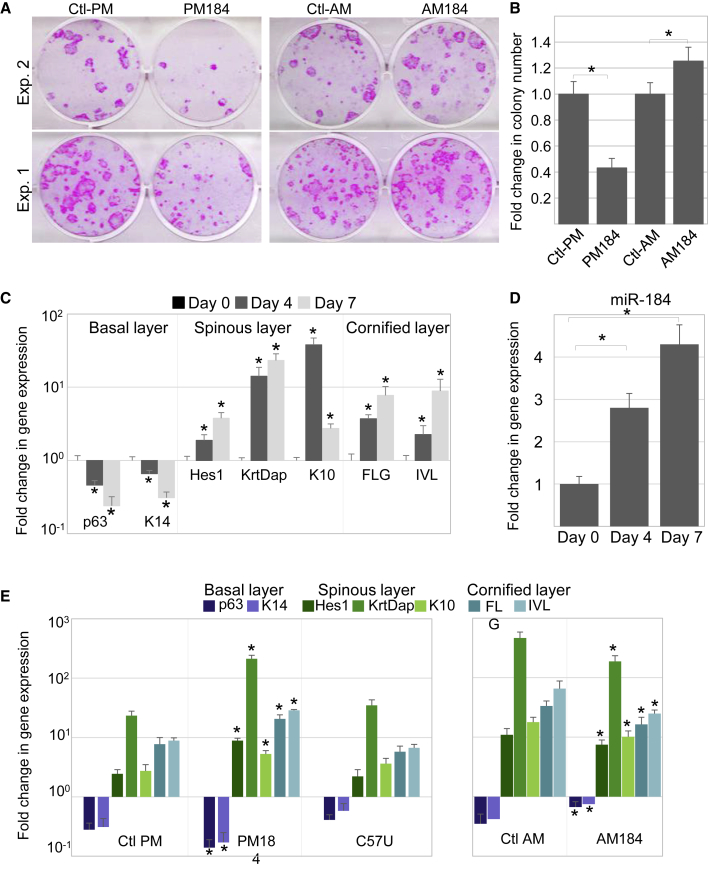
miR-184 Repressed the Clonogenic Potential and Accelerated Epidermal Differentiation (A and B) Primary human foreskin KCs were transfected with pre-miR-184-mimic (PM184) or anti-miR antagonist (AM184) or appropriate controls (Ctl-PM and Ctl-AM, respectively) and then subjected to the clonogenicity test. Colonies were visualized by Rhodamine staining (A, examples of two experiments are shown) and quantification by computerized analysis is shown in (B). (C and D) Primary human KCs were induced to differentiate/stratify by high calcium, and quantitative real-time PCR analysis of the indicated genes was performed to validate differentiation efficiency (C) and TaqMan assay to test the expression of miR-184 (D). (E) Primary KCs were transfected with PM184 mimic, or with disease-causing pre-miR-184-mutant (C57U) mimic or antagonist (AM184), or the appropriate control oligonucleotides, and then subjected to calcium-induced stratification/differentiation. Real-time PCR analysis was performed to evaluate the effect of transfection on differentiation. Data shown are means ± SD from three independent experiments. ^∗^p < 0.05 statistically significant by Student's t test.

To test the potential effect of miR-184 on long-term proliferation of stem/progenitor cells in human cells, foreskin KCs were transfected with pre-miR-184 mimic (PM184), anti-miR-184 antagonist (AM184), or control oligonucleotides (Ctl-PM and Ctl-AM, respectively). Forty-eight hours later, transfectants were seeded at low density, and their colony formation ability was examined following 2–3 weeks of growth in culture. As shown in Figures 4A and 4B, the number and size of the clones was largely reduced following PM184 transfection, while transfection with AM184 antagonist resulted in enhanced clonogenic potential. The involvement of miR-184 in SC regulation and differentiation was further tested by a calcium stratification/differentiation assay. KC differentiation was validated by real-time PCR analysis, showing a decrease in stem/progenitor cell markers and increase in differentiation markers over time (Figure 4C). In line with the expression pattern of miR-184 *in vivo*, differentiation was correlated with an elevation in the levels of expression of miR-184 (Figure 4D). Forced expression of PM184 mimic induced a decrease in markers of basal layer cells and an increase in markers of differentiation, compared with control transfection, while AM184 antagonist displayed a reciprocal effect (Figure 4E). Interestingly, transfection with pre-miR that mimics the disease-causing miR-184 (C57U) had no significant effect on differentiation (Figure 4E), suggesting a loss-of-function mechanism. Altogether, these data suggest that miR-184 inhibits SC/progenitor clonogenic potential and induces differentiation in human KCs.

### Identification of miR-184 Target Genes in the Epidermis

**Figure 5:**
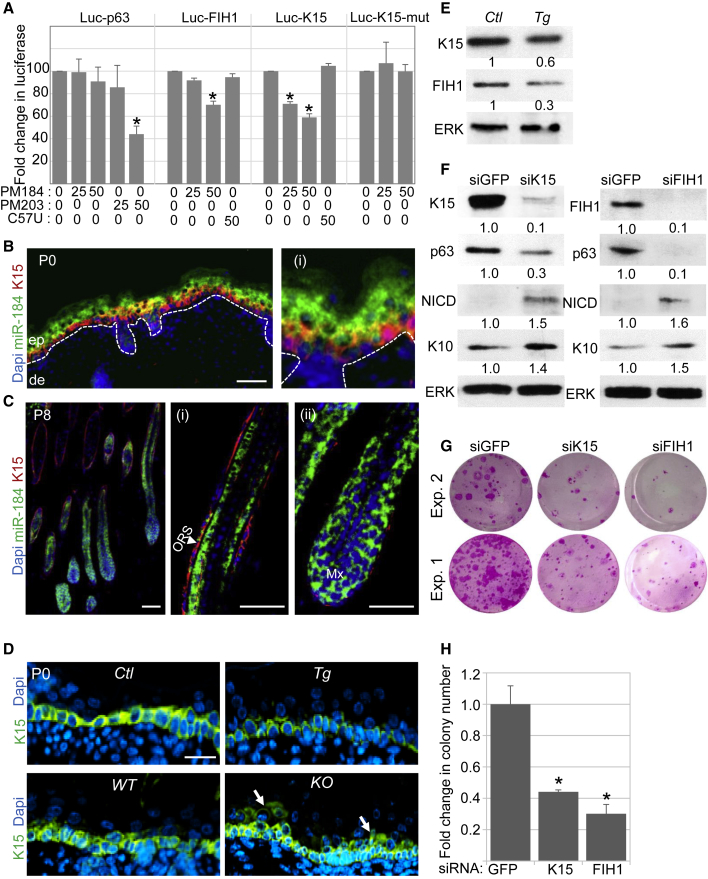
FIH1 and K15 Are Direct Targets of miR-184 that Maintain Epidermal Stemness *In Vitro* (A) HEK293 cells were co-transfected with the indicated concentration (nM) of pre-miR-184 (PM184), disease-causing pre-miR-184^C57U^ mutant (C57U) or pre-miR-203 (PM203), or control oligonucleotides together with luciferase plasmids containing the 3′UTR of the indicated genes downstream to the luciferase encoding sequence. Cells were lysed 24 hr later and the luciferase assay was performed as detailed in the Experimental Procedures. (B and C) *In situ* hybridization of miR-184 coupled with K15 immunostaining on mouse head cryosections at P0 (B) and P8 (C). Higher magnification pictures of (C) are shown in (i) and (ii). (D) Immunofluorescent staining of K15 on mouse sections of newborn mice of the indicated genotypes. Arrows show abnormal expression of K15 in supra-basal cells of KO mice. (E) Primary KCs of the indicated genotypes were lysed and subjected to western blot analysis for K15, FIH1, or ERK as loading control. (F) KCs were transfected with endoribonuclease-prepared silencing RNAs against K15 (siK15) or FIH1 (siFIH1) or GFP (siGFP) as control. Lysates were prepared 48 hr later for immunoblotting against the indicated proteins. (G and H) Primary human foreskin KCs were transfected with the indicated silencing RNAs and then subjected to the clonogenicity test. Colonies were visualized by Rhodamine staining (G, examples of two experiments are shown), and quantification by computerized analysis is shown in (H). Data shown are means ± SD from three independent experiments. ^∗^p < 0.05 statistically significant by Student's t test. Quantification of western blot analysis from at least three independent experiments (p < 0.05) is shown at the bottom of each panel. The dashed line in (B) indicates the dermal-epidermal junction. Scale bars are 50 μm for (B) and (C) and 20 μm for (D). de, dermis; ep, epidermis; Mx, matrix; ORS, outer root sheath.

To unravel miR-184 pathways in the skin, we used three algorithms (TargetScan, miRanda, MicroCosm) that identify potential target genes for miRNAs based on complementary sequences of given miRNA to the 3′-untranslated region (3′UTR) of mRNAs. Among other genes, p63, K15, and FIH1 were very interesting candidates that contained a predicted binding site(s) for miR-184 (Figure S5A). p63 and K15 were linked with stemness in the skin and cornea (Ito et al., 2005, Peng et al., 2012, Senoo et al., 2007), and p63 was modulated by miR-184 (Figures 2 and 3). FIH1 has already been reported to be a direct target of miR-184 in glioma (Yuan et al., 2014) and to negatively regulate Notch activity (Peng et al., 2012), but its role in the epidermis is not clear. We thus tested the possibility that miR-184 can directly bind to the 3′UTR of these three genes by the luciferase assay (Figure S5B). HEK293 cells were co-transfected with a plasmid containing a luciferase coding sequence upstream to the 3′UTR of p63 (p63-Luc) and PM184 mimic or non-specific oligonucleotides as control (Ctl-PM). As shown in Figure 5A, only a mild decrease in the luciferase activity was observed in the presence of PM184 mimic. In contrast, miR-203 mimic (PM203), a known direct repressor of p63 (Lena et al., 2008, Yi et al., 2008), showed significant inhibition (Figure 5A). This suggests that miR-184 does not directly inhibit p63, and we conclude that the observed repression of p63 by miR-184 (Figures 2, 3, and 4) was indirect, potentially a consequence of Notch activation. Notably, PM184 inhibited the luciferase activity when co-transfected with Luc-K15 plasmid, while point mutation in the 3′UTR plasmid (Luc-K15-mut, Figure S5B), which disrupted the miR-184-predicted binding site, completely abolished the effect of PM184 (Figure 5A). In line with a previous report (Peng et al., 2012), miR-184 efficiently inhibited luciferase activity when co-transfected with Luc-FIH1. Interestingly, disease-causing C57U-miR-184 mimic did not significantly affect luciferase activity when co-transfected with Luc-K15 or Luc-FIH1 plasmids (Figure 5A). *In situ* hybridization of miR-184 coupled to K15 immunostaining illustrated that miR-184 and K15 are reciprocally expressed in the epidermis and hair follicle (Figures 5B and 5C), with only scarce co-expression in the same cells. In agreement, the levels of K15 significantly decreased upon miR-184 forced expression and increased upon miR-184 ablation (Figures 5D and 5E). Finally, as the available FIH1 antibodies could not faithfully detect FIH1 protein by staining on tissue sections, we performed western blot analysis, which confirmed that miR-184 significantly repressed FIH1 *in vivo* (Figure 5E). Taken together, these results suggest that miR-184 directly represses both K15 and FIH1 in the epidermis *in vivo*.

The repression of FIH1 or K15 could contribute to miR-184-observed functions; however, the role of K15 and FIH1 in the KC proliferation and differentiation is unknown. For efficient and specific gene repression, we used an endoribonuclease-prepared mixture of siRNA against each gene or against green fluorescent protein (GFP) as control. As shown in Figure 5F, repression of either K15 or FIH1 resulted in a decrease in p63 and elevation of early differentiation genes (NICD, K10). Furthermore, similar to the effect of miR-184 on the long-term proliferation of KCs (Figures 4A and 4B), repression of K15 or FIH1 resulted in significant reduction in KC clonogenicity (Figures 5G and 5H). Altogether, these data suggest that by repressing K15/FIH1, miR-184 induces a differentiation program through Notch activation.

### miR-184 Induced Differentiation through Activation of Notch Signals

**Figure 6:**
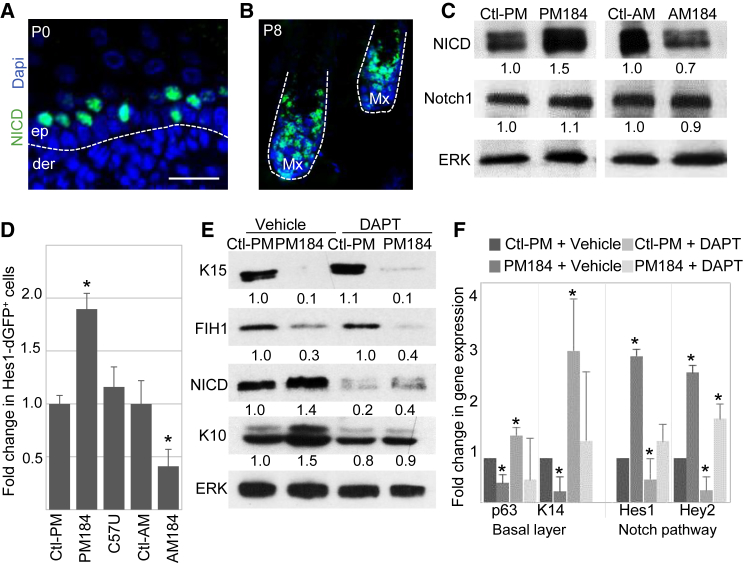
miR-184 Activates the Notch Pathway to Induce KC Differentiation (A and B) Paraffin sections of wild-type mice at the indicated postnatal day were stained for NICD. Scale bars, 50 μm. (C) KCs were transfected with pre-miR-184 mimic (PM184), miR184-antagonist (AM184), or the appropriate control (Ctl-PM and Ctl-AM, respectively) and harvested after 48 hr for western blot analysis of the indicated genes. (D) KCs were co-transfected with a Notch activity reporter plasmid (Hes1-dGFP) and PM184 mimic or miR-184 mutant mimic (C57U) or antagonist (AM184). Forty-eight hours later, cells were trypsinized, and the frequency of GFP-positive cells was quantified by flow cytometry. Data represent the fold increase in GFP-positive cells compared with control transfectants. (E and F) KCs were co-transfected with PM184 or control (Ctl-PM) and, on the next day, treated with the γ-secretase inhibitor that is required for Notch activation (DAPT) or vehicle (DMSO). After 48 hr, cells were subjected to western blot (E) or quantitative real-time PCR (F) analyses of the indicated genes and proteins. Data shown are means ± SD from three independent experiments. ^∗^p < 0.05 statistically significant by Student's t test. Quantification of western blot analysis from at least three independent experiments (p < 0.05) is shown at the bottom of each panel. The dashed line indicates the dermal-epidermal junction in (A) and the hair follicle in (B). de, dermis; ep, epidermis.

**Figure 7:**
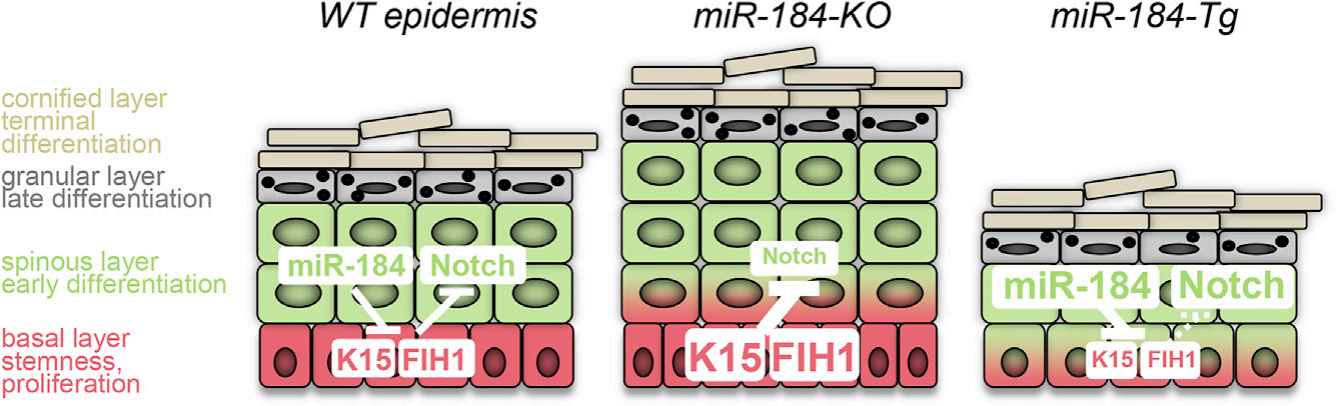
A Working Model miR-184 regulates the balance between basal layer cell proliferation and Notch-dependent differentiation in the epidermis. By targeting K15 and FIH1, miR-184 induces the Notch pathway and thereby represses epidermal stemness, inhibits proliferation, and induces the differentiation program.

In order to examine further the regulation of Notch by miR-184, we performed staining of NICD on wild-type mice (Figures 6A and 6B). In line with a previous report (Blanpain et al., 2006) and similar to miR-184 (Figures 1B, 5B, and 5C), Notch activity was high in the spinous layer cells, which are post-mitotic, and in matrix cells, which are highly proliferative cells. There was no impact on hair growth in miR-184 mutants, suggesting that miR-184/Notch is not involved in the regulation of matrix cell proliferation. We next explored the impact of the miR-184/Notch axis on differentiation. KCs that were transfected with PM184 mimic showed increased levels of NICD while AM184 antagonist had an opposite effect and uncleaved Notch remained unchanged (Figure 6C), suggesting that miR-184 controls Notch at the level of activation. To confirm the effect of miR-184 on Notch transcriptional activity, we used Hes1-dGFP reporter plasmid that contains a destabilized GFP under the promoter of Hes1. Flow cytometry analysis showed that transfection with PM184 mimic resulted in a significant increase in GFP-positive cells, while the miR-184 mutant (C57U) miRNA mimic had an insignificant effect, and the AM184 antagonist had an opposite effect (Figure 6D). Finally, to test whether Notch activation is necessary for miR-184-induced differentiation, transfected KCs were treated with a γ-secretase inhibitor (DAPT) that prevents Notch cleavage and thus the production of active NICD. As shown in Figures 6E and 6F, miR-184-repression of K15 and FIH1 was not reversed by DAPT treatment, indicating that this effect is upstream to Notch and most likely a direct effect. In contrast, miR-184-induced repression of basal layer genes (p63, K14) and enhancement in spinous layer genes (K10, Hes1, Hey2) was attenuated in the presence of DAPT, suggesting that miR-184 acts upstream to Notch and its effect on differentiation depends on Notch activation. Altogether, we conclude that miR-184, through direct repression of K15 and FIH1, induces an exit from epidermal stemness/proliferation and accelerates Notch-dependent differentiation (Figure 7).

## Discussion

Very few studies have described a single miRNA that plays a potent role *in vivo*. In the skin, miR-203 was shown to repress epidermal stemness by targeting p63 (Lena et al., 2008, Yi et al., 2008), while miR-24 induced epidermal cytoskeletal remodeling, differentiation, and cell-cell adhesion (Amelio et al., 2012). In contrast, miR-125b and miR-205 were shown to be positive regulators of HFSCs (Wang et al., 2013, Zhang et al., 2011). The present study shows that miR-184 represses basal layer cell proliferation, inhibits p63, and activates Notch in mouse models. In line, miR-184 repressed clonogenic potential and accelerated differentiation of human epidermal cells *in vitro*. Altogether, these observations suggest that miR-184 induces the commitment to differentiation of basal epidermal cells.

The compartmentalized expression of miR-184 is similar in the different but closely related epithelial tissues that were studied, namely, epidermis, hair follicle, and cornea. miR-184 was low expressed in the SC niche (epidermal basal layer, bulge, and limbus) but mainly expressed by cells that migrated out of the niche (epidermal spinous layer, corneal basal layer, ORS, and matrix of the hair follicle). In the epidermis, the transition from the niche is coupled with an exit from the cell cycle. Thus, the elevation in miR-184 and NICD in the spinous layer is well correlated with the observed inhibition of proliferation by miR-184. Although no phenotype has been found yet in the hair follicle of miR-184-KO and transgenic mice, miR-184 and NICD were both detected in matrix cells that are highly proliferative, suggesting that miR184/NICD may have a cell-context-dependent effect on cell proliferation.

The repression of proliferation and activation of Notch by miR-184 suggests that it may play a role as a tumor suppressor in the epidermis (Nicolas et al., 2003). Indeed, miR-184-deficient mice developed a significantly larger numbers of tumors in a two-stage chemical carcinogenesis model (S.N. et al., unpublished data). In line, the oncogenic miR-205 (Cai et al., 2013) is counteracted by competition with miR-184 (Yu et al., 2008). It was shown that miR-184 interferes with miR-205 repression of the phosphatidylinositol 3-kinase inhibitor, SHIP2 (Yu et al., 2008, Yu et al., 2010), and inhibits cell migration (Yu et al., 2010). These studies link miR-205 with oncogenic properties and its inhibitor, miR-184, as a tumor suppressor. This is in accordance with the role of miR-205 in SC maintenance (Wang et al., 2013) and with our data that miR-184 represses stemness. It is thus conceivable that some of the functions of miR-184 that are described in the current study are mediated by interference with miR-205. Likewise, miR-184 acts as a tumor suppressor in glioma (Malzkorn et al., 2010) and neuroblastoma (Foley et al., 2010). However, it was proposed that miR-184 plays a role as an oncogenic miRNA in head and neck squamous cell carcinoma and in tongue cancer (Wong et al., 2009), suggesting a context-dependent role for miR-184 in cancer.

Our data support a model by which miR-184 regulates Notch activity through repression of K15 and FIH1 (model in Figure 7). FIH1 is a known repressor of HIF1α (Mahon et al., 2001) that has been shown to be a negative regulator of Notch (Zheng et al., 2008). FIH1 hydroxylates asparagine residues of proteins containing an ankyrin repeat domain, including Notch1, IkBa, and p105, while HIF1α is a prominent FIH1 substrate (Cockman et al., 2009). The present study suggests that FIH1 is required to repress Notch activity and maintain proliferative potential, while miR-184, by repressing FIH1, induces the Notch pathway and commitment to differentiation. In agreement, FIH1 is preferentially expressed by LSCs where it regulates cell metabolic status (Peng et al., 2013). In FIH1-null mice, the skin phenotype has not been described yet. Interestingly, a marginal role was found for FIH1 in the classical aspects of HIF regulation such as angiogenesis, and, instead, FIH1-null mice displayed metabolic defects such as reduced body weight, higher insulin sensitivity, and an elevated metabolic rate (Zhang et al., 2010). In line, miR-184 is involved in regulating pancreatic β cell growth and function according to the demand for insulin (Tattikota et al., 2014). Therefore, it would be interesting to examine the role for the miR-184/Notch axis in other tissues where miR-184 is highly expressed and plays an important role, such as the cornea and lens (Hughes et al., 2011, Shalom-Feuerstein et al., 2012) and pancreatic β cells (Tattikota et al., 2014). Although K15 is considered as a marker of quiescent hair-follicle and corneal SCs, the regulation of K15 and the function of K15 remain poorly defined. The expression pattern of K15 is clearly reciprocal to miR-184, strongly suggesting that, as soon as cells depart from the niche, the levels of miR-184 are elevated and, in turn, miR-184 directly represses K15. Evidently, cytokeratins are not only architectural (structural) proteins. Many studies have demonstrated their function in cell polarity (Hobbs et al., 2016), cell proliferation (Depianto et al., 2010), and immunomodulation (Lessard et al., 2013). Previously, it was shown that K15 can compensate for the loss of K14 in specific types of stratified epithelia (Lloyd et al., 1995). In the present study, *in vitro* knockdown of K15 significantly repressed long-term proliferation and enhanced differentiation of human KCs (Figures 5F–5H), suggesting that K15 is important for epidermal homeostasis. miRNAs may have many target genes in a given cellular context. Accordingly, the function of a specific miRNA is mediated by a combinatorial modulation of a set of factors. The data presented here suggest that miR-184 regulates the balance between epidermal proliferation and differentiation through the repression of K15 and FIH1. However, it is likely that other, yet unknown, miR-184-target genes are involved in this process.

In line with a previous study by Ryan et al. (2006), the levels of miR-184 were significantly higher in the cornea compared with the epidermis and heart (two orders of magnitude, see Figure S2H). Indeed, point mutations in miR-184 are linked with autosomal-dominant blinding eye diseases (Farzadfard et al., 2016, Hughes et al., 2011, Lechner et al., 2013, Iliff et al., 2012). These patients suffer from corneal abnormalities and early onset of anterior cataract (lens opacification). We found that miR-184 is expressed at the highest levels in the corneal and lens epithelia, high in the hair follicle, and moderately in the epidermis. To our knowledge, no skin defects were reported in these patients. miR-184-*KO* mice displayed corneal stromal thinning, which is in line with keratoconus found in these patients (to be described elsewhere). Interestingly, the C57U mutation that is located to the seed sequence itself (Hughes et al., 2011) resulted in loss-of-function most likely due to reduced complementary to *bona fide* miR-184 target genes such as FIH1 and K15 (Figure 5A), and failure to induce the Notch pathway and differentiation (Figures 4E and 6D). Future studies will be needed to define the effect of miR-184 mutation on FIH1, K15, and on cell proliferation and differentiation in corneal/lens development, homeostasis, and pathology.

## Experimental Procedures

### Animals Generation and Care

Experiments were performed in accordance with the guidelines and approval of the local ethical committee (IL-066-05-13). The miR-184-*KO* mouse model (C57BL/6N) was generated by Phenomin-iCS, Strasbourg. BD10 (MCI-C57BL/6N Tac) embryonic SCs were electroporated with a construct shown in Figure S1. Five positive clones were selected out of 372 screened, and following successful blastocyst injection, germline transmission and *in vivo* excision of the *Neo* cassette by FRP transgene were achieved. Conditional loxP-miR-184 mice were crossed with *ROSA26-Cre* transgene to generate miR-184-*HT*. Mice were genotyped with forward (5′ ACT GAA CAT TAT TTC ATG GGC CGG G) and reverse (5′ AAC TAC AAC TGT TTG GCT AGC AGG GTG) primers for the knockout allele or with an alternative reverse primer (5′ CGC TGA GAC CTT GTG ATA AAC CGT T) for the amplification wild-type allele. Chi-squared test was performed by GraphPad online software (http://graphpad.com/quickcalcs/chisquared1/).

*KRT14-rtTA* (genetic background FVB) was purchased from (008099, The Jackson Laboratory, Bar Harbor, USA) and crossed with previously described *TRE-miR-184*^*Tg*^ (referred to hereafter as *miR-184*^*Tg*^, genetic background C57BL/6J) (Tattikota et al., 2014). Dox (TD.01306, Harlan) was administered in the food from E12. For genotyping, K14-rtTA-specific primers were forward 5′-GTC CGA TGG GAA AGT GTA GCC TG-3′ and reverse 5′ TTT CTT CTT TAG CGA CTT GAT GC-3′ and *miR-184*^*Tg*^ primers were forward 5′ TGC TGA AGA GTG GCC TGC TAG G and reverse 5′ CTC CTC CTC ACG TCC TGT GGT A.

### Tissue Processing and Staining

Analysis included tissues obtained from three to five mice per genotype from two or more litters. For immunofluorescent staining, tissues were dehydrated and then stained as detailed previously (Amitai-Lange et al., 2015). Primary antibodies were rabbit anti-K10 1:400 (Covance, PRB-159P), mouse anti-K14 1:200 (Millipore, CBL197), mouse anti-FLG 1:75 (Abcam, AB31356), mouse anti-P63 1:100 (Santa Cruz, sc-8431), rabbit anti-Ki67 1:100 (Santa Cruz, sc-7846), mouse anti-K15 1:200 (Santa Cruz, sc-47697), and goat anti-FIH1 1:100 (Santa Cruz, sc-26219). *In situ* hybridization was performed as described previously (Shalom-Feuerstein et al., 2012). To quantify the percentage of Ki67-positive cells among DAPI-positive epidermal basal layer cells, a total of 500 cells of 5 different regions on 5 different slides were manually counted and quantified using ImageJ. Epidermal thickness was measured by using the line selection tool in ImageJ 1.48v using 10 different skin regions in each slide and a total of 30 regions were measured. Similarly, to determine the amount of K14- and K10-positive cells for each genotype, nuclei of DAPI and K10 or K14 co-stained cells were counted in epidermal fields that were defined for the length of the dashed lines indicating the dermal-epidermal border.

### Cell Culture, Transfections, and *In Vitro* Assays

KCs and HEK293 were grown as previously described (Lena et al., 2008). esiRNA reagents were from Sigma-Aldrich and miRNA mimic/inhibitor were from Ambion. Hes1-dGFP reporter plasmid was a kind gift from C. Marcello (Rios et al., 2011). For transfections, we used miRNA mimics and antagonists (50 nM), esiRNA (15 nM), or Hes1-dGFP (2 μg) with Lipofectamine RNAiMAX reagent (Invitrogen). The clonogenicity test, reporter assay, and calcium differentiation were as described (Shalom-Feuerstein et al., 2012). The P63-3′UTR vector was a kind gift from G. Melino and E. Candi, and FIH1-3′UTR was a kind gift from M. Buhler and O. Lapaire (Lalevee et al., 2014). The epidermis was isolated from back skin of newborn mice; tissues or cultured cells were lysed and sonicated in RIPA buffer (Shalom-Feuerstein et al., 2012) and further prepared as previously described (Shalom-Feuerstein et al., 2004, Shalom-Feuerstein et al., 2008). Real-time PCR analysis was as reported by Shalom-Feuerstein et al. (2012).

### Statistical Analysis

For all measurements, the error bars represent the SDs. Student's two-tailed t test was used to determine the respective statistical significance. Probability (p) values are given in the figure legends; p < 0.05 was considered statistically significant.

## Author Contributions

S.N. and F.L. designed and performed experiments, analyzed data, prepared the figures, and participated in the manuscript writing; D.P. designed and performed experiments, analyzed data, and prepared the figures; S.B., A.A., L.S., W.N., E.A., and A.A.-L. performed experiments and prepared the figures; S.G.T. and M.N.P. contributed with transgenic mice; M.R. discussed the results and contributed to the editing of the manuscript; D.A. and R.S.-F. provided financial support, designed experiments, discussed the results, and wrote the manuscript. All authors approved the manuscript.

## Supplemental Information

Document S1. Figures S1–S5 and Table S1

Document S2. Article plus Supplemental Information
