# Supplementary material for: microRNA-184 Induces a Commitment Switch to Epidermal Differentiation
Source: Stem Cell Reports. 2017 Nov 30;9(6):1991–2004. doi: 10.1016/j.stemcr.2017.10.030 (PMC5785777; doi:10.1016/j.stemcr.2017.10.030)
Supplement: Document S1. Figures S1–S5 and Table S1 [file mmc1.pdf]

Stem Cell Reports, Volume *9*

Supplemental Information

microRNA-184 Induces a Commitment Switch to Epidermal Differentiation

Sara Nagosa, Friederike Leesch, Daria Putin, Swarnabh Bhattacharya, Anna Altshuler, Laura Serror, Aya Amitai-Lange, Waseem Nasser, Edith Aberdam, Matthieu Rouleau, Sudhir G. Tattikota, Matthew N. Poy, Daniel Aberdam, and Ruby Shalom-Feuerstein

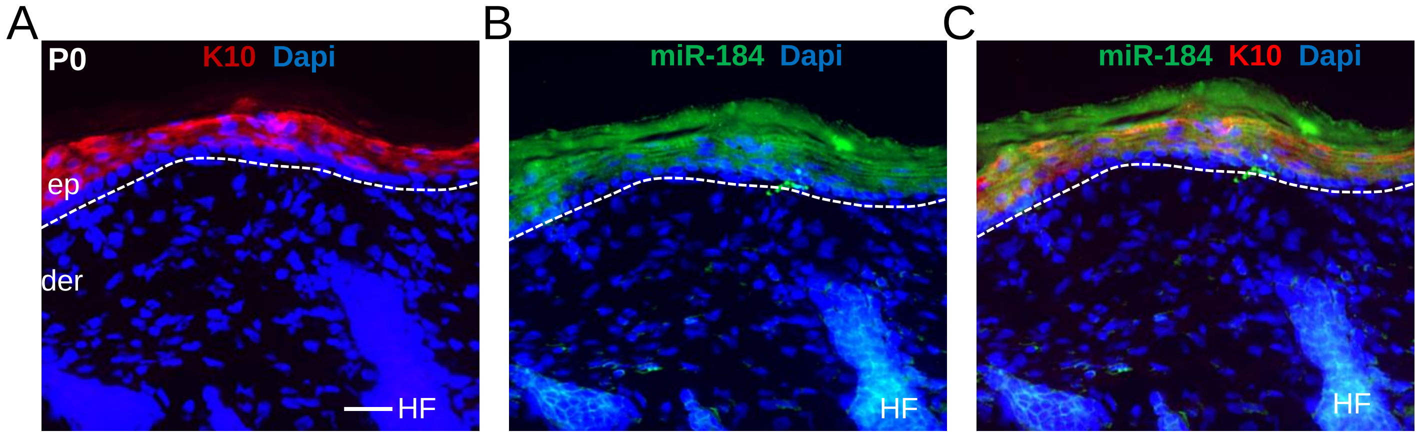

Figure S1 (Related to figure 1). Co-expression of miR-184 and K10. In situ hybridization coupled with immunofluorescent staining of K10 was performed on tissue sections of new born (P0) wild type mouse. Each channel and Dapi counter stain is shown alone (A-B). Merge of the three channels is shown in C. Dashed line indicates the dermal-epidermal junction. Scale bar 50 μm. Abbreviations: de, dermis; ep, epidermis; HF, hair follicle.

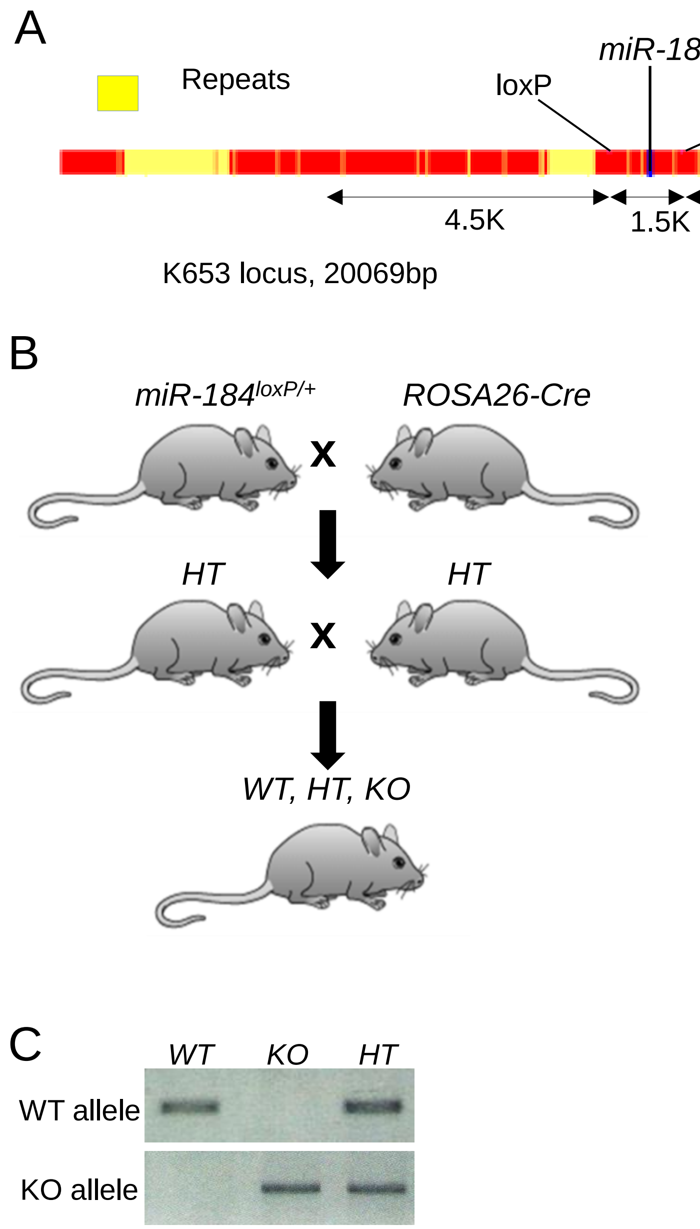

D

	WT	*HT*	*KO*	Total
**No. of mice (%)**	81 (31.8%)	109 (42.8%)	65 (25.5%)	255

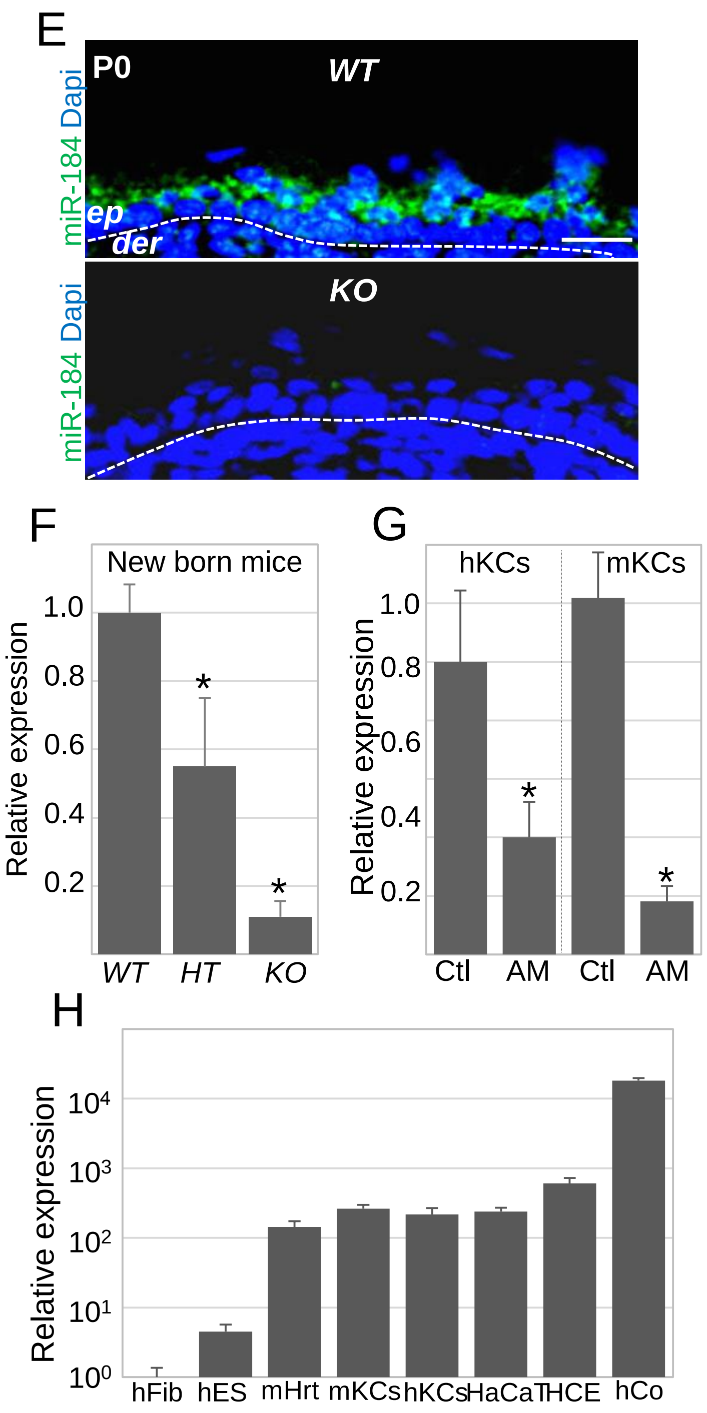

Figure S2 (Related to figure 2). Generation of miR-184 *KO* mice and comparative analysis of miR-184 expression. A. Schematic representation of the strategy for generating conditional miR-184-floxed allele. The 5' homology arm was of ~4.5kb, 3' homology arm of ~3.5kb and floxed fragment was of ~1.5kb. B. Schematic illustration for the generation of miR-184-*KO*. Genotyping verified the deletion of miR-184 gene (C). D. Summary of the numbers of mice that were obtained for each genotype in this study (n=255). (E) In situ hybridization of miR-184 on skin sections of the indicated genotypes. Scale bars 50 µm. (F) RNA was extracted from the epidermis of the indicated genotypes at P0 and subsequently miR-184 TaqMan real time PCR assay was performed. (G) Wild type primary keratinocytes of human (hKCs) or mouse (mKCs) were transfected with control oligo (Ctl) or with anti-miR-184 antagonist (AM) and then lysed and miR-184 expression was examined by TaqMan real time PCR assay. (H) The expression of miR-184 was examined by TaqMan assay in RNA samples that were extracted from the indicated cells. Abbreviations: human fibroblasts (hFibs); human embryonic stem cells (hES); newborn murine heart tissue extract (mHrt); newborn murine epidermal keratinocytes (mKC); human foreskin keratinocytes (hKC); immortalized human keratinocytes (HaCaT); immortalized human corneal epithelial cells (HCE).

A

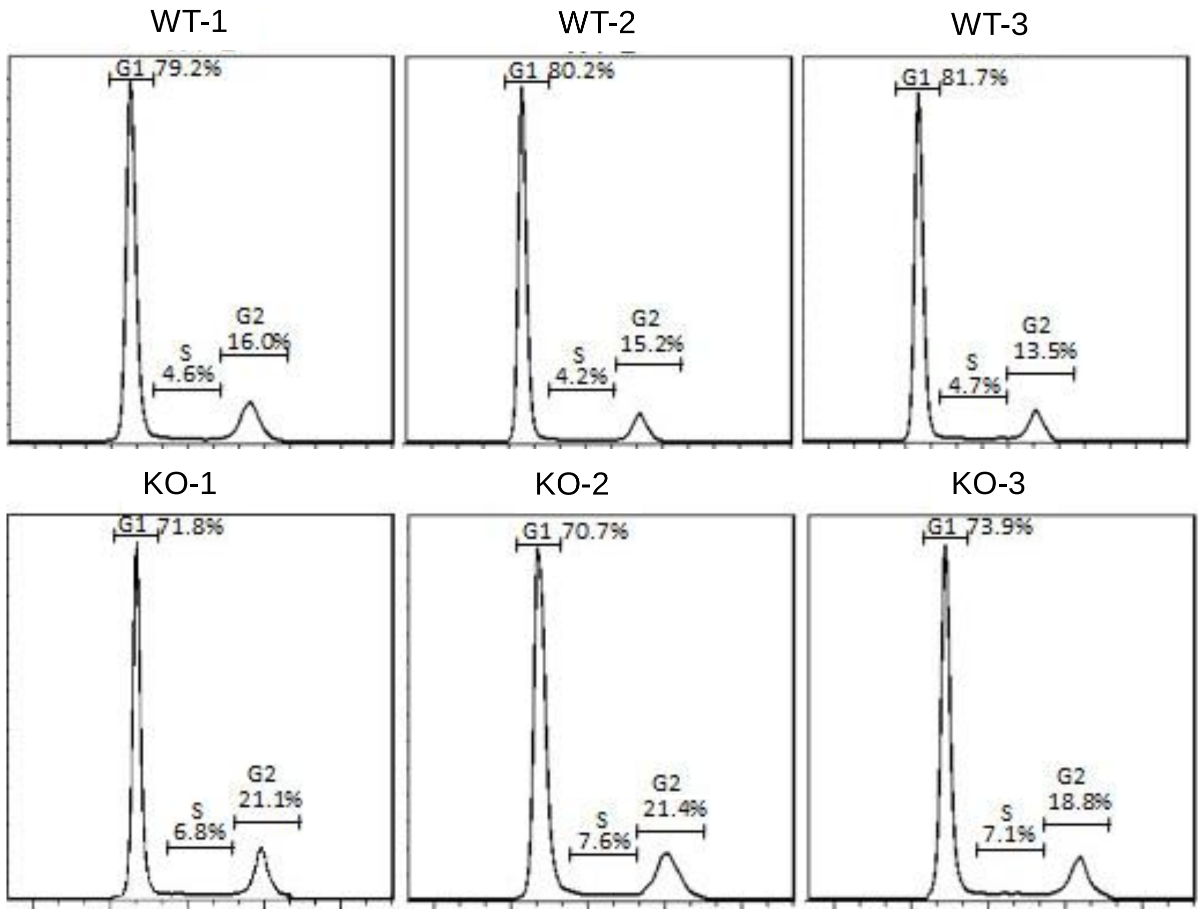

B

Genotype	G1	S	G2/M
WT	80.37±1.26	4.50±0.26	14.90±1.28
KO	72.13±1.63	7.17±0.40	20.43±1.42

Figure S3 (Related to figure 2). Cell cycle analysis of WT and KO epidermal cells. Keratinocytes were isolated from the epidermis of newborn mice and subjected to cell cycle analysis by flow cytometry. Analysis from samples of three different mice for each genotype is shown in A, and the average percentage of each population is shown in B. Data represents means ± SD.

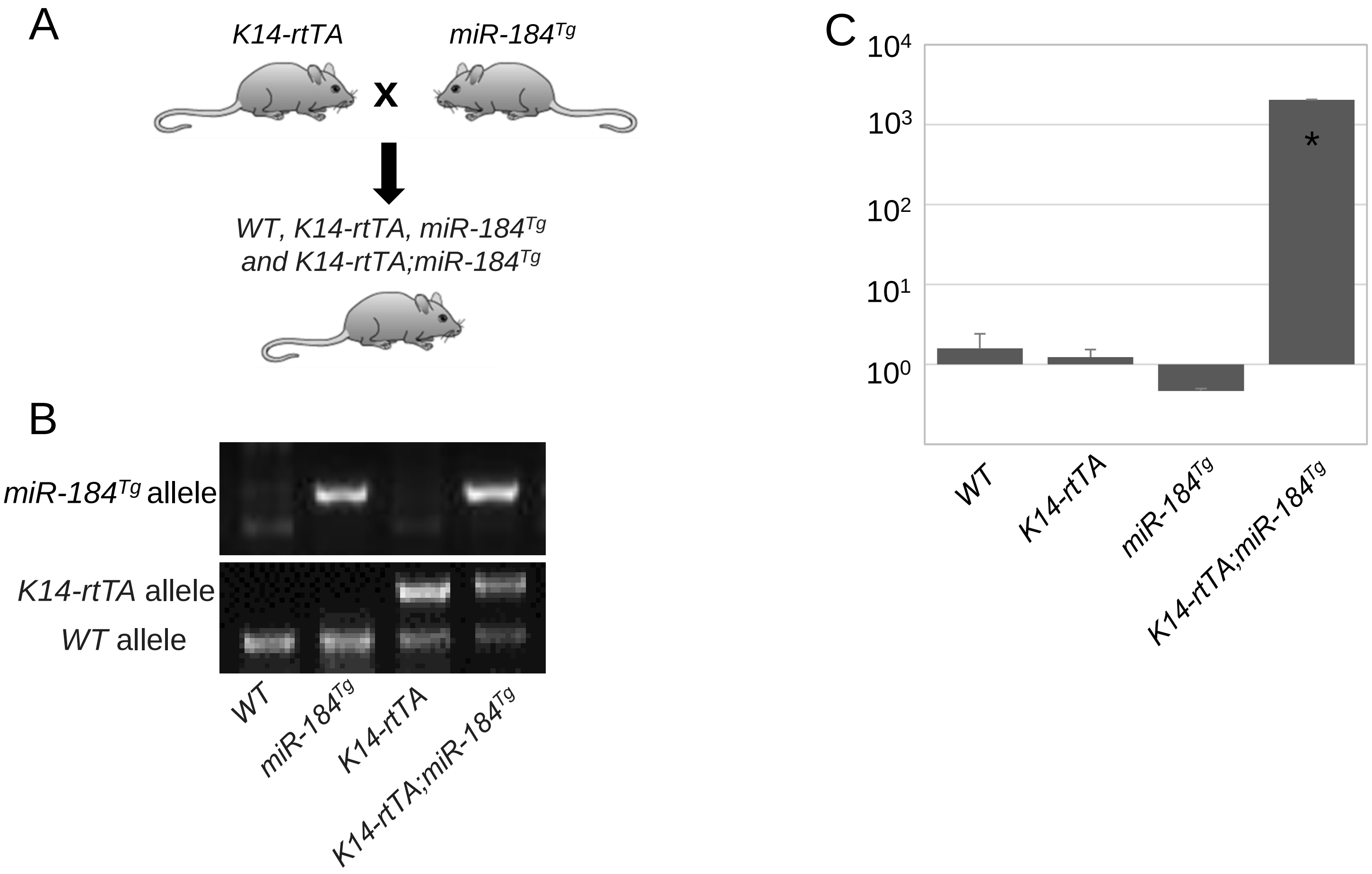

Figure S4 (Related to figure 3). Generation of inducible miR-184 transgenic expression under K14 promoter. A. Hemizygous *miR-184^{Tg}* mice were crossed with hemizygous mice containing the reverse tertracycline transactivator (*rtTA*) transgene under K14 promoter (*K14-rtTA*) (Jackson Laboratory) resulting in the indicated genotypes that were validated by PCR (B). C. Pregnant females were induced with the tetracycline analogue doxycycline (Dox) in food from E12. TaqMan assay for miR-184 confirmed the induced expression only in double transgenic mice (*K14-rtTA*;*TRE-MIR-184^{Tg}*) that served as the experimental group while *K14-rtTA* served as controls.

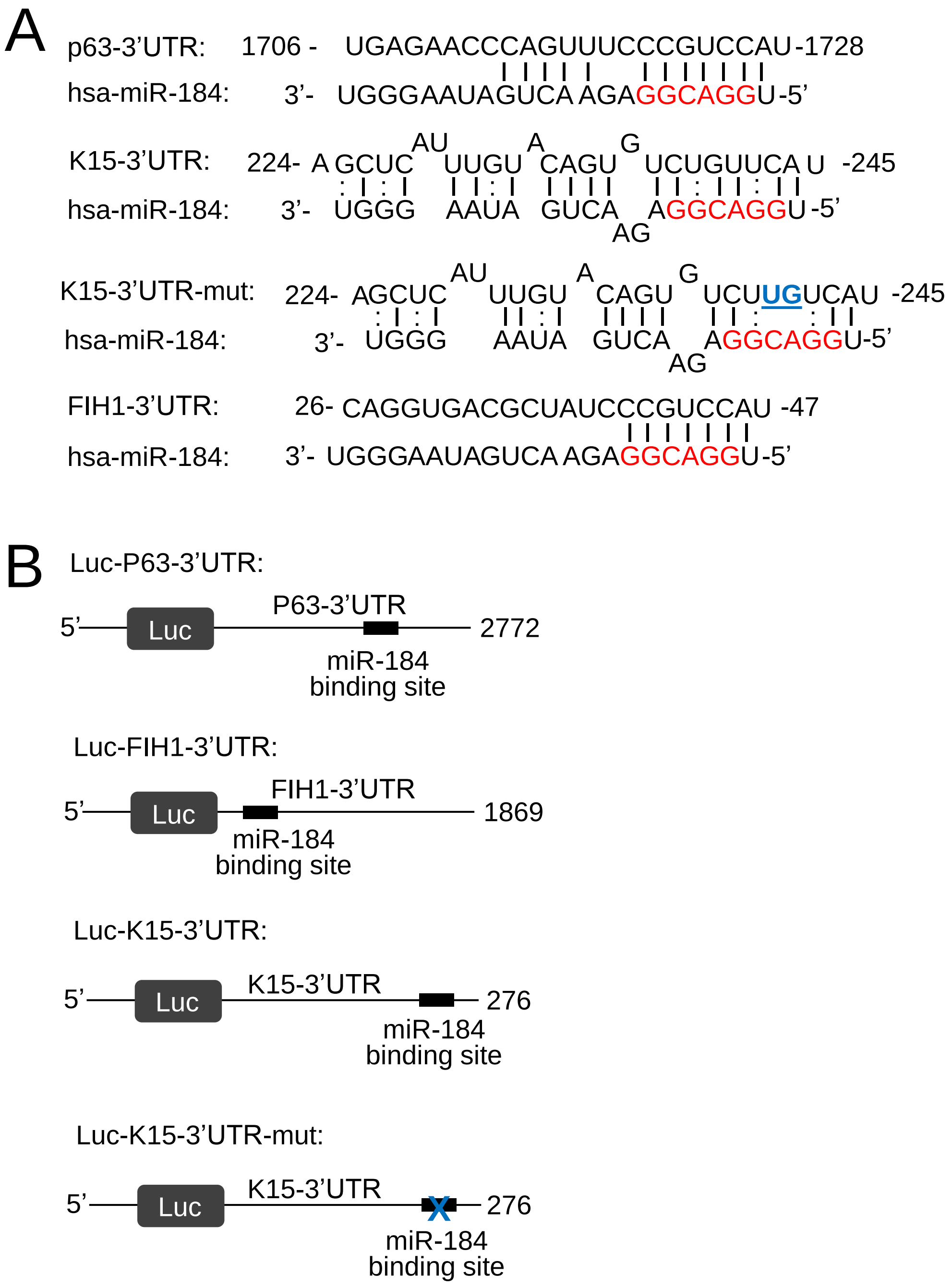

Figure S5 (Related to figure 5). **Study of miR-184 potential target genes and constructs.** A. In silico predication for miR-184 target interaction with 3'UTR of K15, p63 and FIH1. Positions of the potential binding sites of the seed sequence of miR-184 are indicated in red. Illustration of the mutation performed in the fragment of K15-3'UTR (K15-3'UTR-mut) which was cloned into luciferase plasmid (Luc-K15-3'UTR-mut, illustrated in B). The annotated underlined nucleotides in blue, is the point mutation from GU to UG. B. Schematic representation of luciferase (Luc) constructs containing the 3'UTR of the K15 (K15-Luc) or FIH1 (FIH1-Luc) that was cloned downstream Luc gene. Potential miR-184 binding sites in the 3'UTR of K15 and FIH1 constructs (62) are shown.

Primer ID	Sense primer (5'-3')	Antisense primer (5'-3')
**human**		
FIH1	TTCCCGACTAGGCCCATTC	CAGGTATTCAAGGTCCCATTTCA
36B4	CTCCTGTCATCAACGTACAC	CACTCCCCTTAGGGAGCCTTG
FLG	CTGTGGTCATTCATGGAGTGG	CCCTAGAAGGGCTAATGTGTGA
HES1	CCTGTCATCCCCGTCTACAC	CACATGGAGTCCGCCGTAA
HEY2	GCCCGCCCTTGTCAGTATC	CCAGGGTCGGTAAGGTTTATTG
IVL	TCCTCCAGTCAATACCCATCAG	CAGCAGTCATGTGCTTTTCCT
K10	ATGTCTGTTCGATACAGCTCAAG	CTCCACCAAGGGAGCCTTTG
K14	TGAGCCGCATTCTGAACGAG	GATGACTGCGATCCAGAGGA
K15	GACGGAGATCACAGACCTGAG	CTCCAGCCGTGTCTTTATGTC
KrtDap	CCATTGAGAATTATGCGTCACGA	CAATTTGTCGATGTTCAGGAACG
p63	GTCATTTGATTCGAGTAGAGGGG	CTGGGGTGGCTCATAAGGT
**mouse**		
FIH1	GTCCCAGCTACGAAGTTACAGC	CAGTGCAGGATACACAAGGTTT
36B4	AGCTTCGGCACATATTTCATCTG	CGTTCACTCCCATGACAAACA
FLG	CTAGAGGGCATGAGTGTAGTCA	CAAGACTGGACAGTTGGCTGG
HES1	GATAGCTCCCGGCATTCCAAG	GCGCGGTATTTCCCCAACA
HEY2	AAGCGCCCTTGTGAGGAAAC	GGTAGTTGTCGGTGAATTGGAC
IVL	ATGTCCCATCAACACACACTG	TGGAGTTGGTTGCTTTGCTTG
K10	CGAAGAGCTGGCCTACCTAAA	GGGCAGCGTTCATTTCCAC
K14	CAGTATCCGATCTCTTCATGCG	GGGCTCACAGAAGGTTTCCTG
K15	CTGTTTCCGGCCTAAGCAATA	CCTGAGAGCGAATGCCAGA
p63	CACCTGGACGTATTCCACCG	CATGGCACGGATAACAGCG

Table S1: A list of primers that were used for real time PCR.
